# Supplementary material for: Hybrid Models and Biological Model Reduction with PyDSTool
Source: PLoS Comput Biol. 2012 Aug 9;8(8):e1002628. doi: 10.1371/journal.pcbi.1002628 (PMC3415397; doi:10.1371/journal.pcbi.1002628)
Supplement: Text S4 — Complete source code for the PyDSTool package (version 0.88.120504). Includes API documentation and help files linking to web pages. This file is identical to the current public release on Sourceforge.net. (ZIP) [file pcbi.1002628.s004.zip › PyDSTool/html/scipy.integrate.vode-module.html]

xml version="1.0" encoding="ascii"?


scipy.integrate.vode


| Home | Trees | Indices | Help | PyDSTool |
| --- | --- | --- | --- | --- |

Package scipy ::
Package integrate ::
Module vode

[hide private]

[frames] | no frames]

# Module vode

```
This module 'vode' is auto-generated with f2py (version:2_5540).
Functions:
  y,t,istate = dvode(f,jac,y,t,tout,rtol,atol,itask,istate,rwork,iwork,mf,f_extra_args=(),jac_extra_args=(),overwrite_y=0)
  y,t,istate = zvode(f,jac,y,t,tout,rtol,atol,itask,istate,zwork,rwork,iwork,mf,f_extra_args=(),jac_extra_args=(),overwrite_y=0)
.
```

---

**Version:**
$Revision: $


|  |  |
| --- | --- |
| Variables | [hide private] |

dvode = `getattr(_vode, 'dvode', None)`

zvode = `<fortran object at 0x64bde8>`

| Home | Trees | Indices | Help | PyDSTool |
| --- | --- | --- | --- | --- |

|  |  |
| --- | --- |
| Generated by Epydoc 3.0.1 on Fri May 4 15:24:06 2012 | http://epydoc.sourceforge.net |
